# Burden of COVID-19 in the Pediatric Population at Hospital Central de Maputo, Mozambique, October 2020 to October 2022

**DOI:** 10.3390/v16071112

**Published:** 2024-07-11

**Authors:** Adilson Fernando Loforte Bauhofer, Emerson Miranda, Édio Ussivane, Assucênio Chissaque, Luciana António, Fernanda Campos, Ramígio Pololo, Fátima Iahaia, Aline Gatambire, Fátima Ráice, Marlene Djedje, Judite Salência, Plácida Maholela, Luzia Gonçalves, Osvaldo Inlamea, Nilsa de Deus

**Affiliations:** 1 Instituto Nacional de Saúde, Marracuene 3943, Mozambiquenilsa.dedeus@ins.gov.mz (N.d.D.); 2 Instituto de Higiene e Medicina Tropical, Universidade Nova de Lisboa, Rua da Junqueira 100, 1349-008 Lisboa, Portugal; luziag@ihmt.unl.pt; 3 Global Health and Tropical Medicine, Instituto de Higiene e Medicina Tropical, Universidade Nova de Lisboa, Rua da Junqueira 100, 1349-008 Lisboa, Portugal; 4 Centro de Estatística e Aplicações, Universidade de Lisboa, 1749-016 Lisboa, Portugal; 5 Z-Stat4life, Espaço Cowork Baldaya, Palácio Baldaya, Estrada de Benfica N° 701ª, 1549-011 Lisboa, Portugal; 6 Departamento de Ciências Biológicas, Faculdade de Ciências, Universidade Eduardo Mondlane, Av. Julius Nyerere-Campus Universitário, Maputo 257, Mozambique

**Keywords:** children, COVID-19, death, hospital, Mozambique

## Abstract

The epidemiology and characteristics of SARS-CoV-2 in the hospitalized Mozambican pediatric population are scarce. We aimed to assess the burden of COVID-19 in the pediatric population at Hospital Central de Maputo and identify comorbidities and factors associated with death among hospitalized COVID-19 cases. A cross-sectional study was conducted from October 2020 to October 2022. Available records were retrieved from admission books. Univariate and bivariate analyses were reported to describe the sample characteristics. The frequency of pediatric cases admitted with COVID-19 was 0.6% (95% confidence interval (CI): 0.5–0.6; 364/63,753), and the frequency of pediatric cases hospitalized with COVID-19 was 2.5% (95% CI: 2.2–2.9; 173/6807). The monthly frequency of pediatric cases admitted and hospitalized with COVID-19 ranged from 0.1% to 5.4% and from 0.2% to 42.1%, respectively. In children hospitalized with COVID-19, underweight was the most observed comorbidity (17.4%; 19/109); death was observed in 30% (95% CI: 22.2–39.1; 33/110), and it was significantly higher in underweight children than in non-underweight children (61.5% [8/13] vs. 21.3% [16/75]; *p*-value = 0.005). Given the heightened risk of mortality among undernourished children compared to non-undernourished children, vaccination for COVID-19 should be prioritized for undernourished children.

## 1. Introduction

The severe acute respiratory syndrome coronavirus 2 (SARS-CoV-2) that causes coronavirus disease 2019 (COVID-19), evolved into a public health emergency of international concern in January 2020 and was declared a pandemic in March 2020 [1]. To prevent the spread of the pandemic and the latter overcapacity of health facilities to identify and treat COVID-19 cases, non-pharmacological interventions were implemented. These included washing hands or using an alcohol-based solution, the use of face masks, staying at home or self-isolation for infected individuals, and compliance with social distancing [2].

Early evidence suggested that children had lower susceptibility to SARS-CoV-2 infection compared to adults [3]. However, with the emergence of different SARS-CoV-2 variants, COVID-19 morbidity and mortality showed different trends. In South Africa, hospitalization of COVID-19 cases in patients less than 18 years old was 10.3%, and mortality was 0.1% [4], while in the United States of America, 1.8% of children up to 18 years old with COVID-19 were hospitalized without complications, 1.9% experienced admission to intensive care and/or artificial respiratory support, and 0.02% died [5].

Pre-existing comorbidities exacerbate disease severity and mortality [6,7,8]. The burden and geographical distribution of morbidities can present heterogeneous characteristics in different settings; thus, children’s risk of developing COVID-19 depends on their geographic location [6]. For instance, in Pakistan, cardiovascular disease was mostly associated with severe outcomes [9], while in African countries such as the Democratic Republic of Congo, Ghana, Kenya, South Africa, and Uganda in the first year of the COVID-19 pandemic, cancer, hypertension, chronic kidney disease, HIV, and active tuberculosis were associated with severe outcomes [10]. On the other hand, in the United States of America, disease severity in hospitalized children and adolescents was associated with obesity/type 2 diabetes mellitus and cardiovascular, neuromuscular, and pulmonary diseases [11].

In Mozambique, a low-income African country, the burden of COVID-19 on the pediatric admission was not fully assessed. Available evidence gathered in approximately two months showed a 5.4% mortality rate among infants with COVID-19 from a breastfeeding ward during the second wave [12]. Therefore, we propose evaluating the COVID-19 burden and its monthly distribution in pediatric outpatients and inpatients (i.e., hospitalized patients) as well as describing comorbidities and factors associated with death in hospitalized children with COVID-19 over two consecutive years.

## 2. Materials and Methods

This cross-sectional study was conducted at Hospital Central de Maputo, a public quaternary hospital located in Maputo City, in the Southern region of Mozambique. We screened all admission books from the Pediatric Department of Hospital Central de Maputo since October 2020 to October 2022. Records from March 2020 (the period in which the first COVID-19 case was detected in Mozambique [13]) until September 2020 were not available.

Cumulative data from admissions were stratified into non-COVID-19 and COVID-19 suspected/confirmed cases. COVID-19 suspected/confirmed cases included all children with a SARS-CoV-2 laboratory diagnosis or children with COVID-19-suggestive symptoms and a collected sample for laboratory confirmation.

The cumulative COVID-19 suspected/confirmed cases were further stratified into hospitalized and non-hospitalized patients. The unique identification numbers of all children hospitalized with COVID-19 were retrieved from the admission books, and their individual clinical files were traced to collect sociodemographic data (e.g., sex and age), clinical characteristics (e.g., fever, difficulty breathing, comorbidities), and hospitalization outcomes. Individual data were collected using an Open Data Kit application on a secure tablet or smartphone.

Underweight and stunting were assessed using the z-scores, which were calculated with WHO Anthro software version 3.2.2 for children under five years and with the WHO Anthro Plus software version 1.0.4 for children aged five years or older. Children with z-scores below minus two were labeled as undernourished after excluding z-score flag values from the data; otherwise, they were categorized as non-undernourished.

Data analysis was conducted using R (Austria, Vienna) software version 4.1.0, and graphics were built in Microsoft Office Excel. The relative and absolute frequencies of the monthly distribution of admitted and hospitalized suspected/confirmed COVID-19 cases were determined. Descriptive statistics were used to describe the sociodemographic and clinical characteristics and outcome of the children hospitalized with COVID-19. Binomial proportions were estimated with 95% confidence intervals (CI) using Wilson’s method instead of Wald’s method [14].

A case-wise deletion procedure was used to allow for complete analysis of all eligible cases with a valid response in each variable. Cross-tabulations between sociodemographic and clinical characteristics and outcome status were made, and chi-square or Fisher’s exact tests were used according to the expected counts for qualitative variables. The Mann–Whitney U test was used for quantitative variables (or ordinal variables) in case of the failure of the assumptions of Student’s *t*-test for two independent samples. *p*-values less than 5% were considered statistically significant.

This study was approved by the National Bioethics Committee for Health from Mozambique (IRB00002657; reference number 71/CNBS/22).

## 3. Results

### 3.1. Sample Characteristics and COVID-19 Burden in Pediatric Admission and Hospitalized Cases

**Figure 1:**
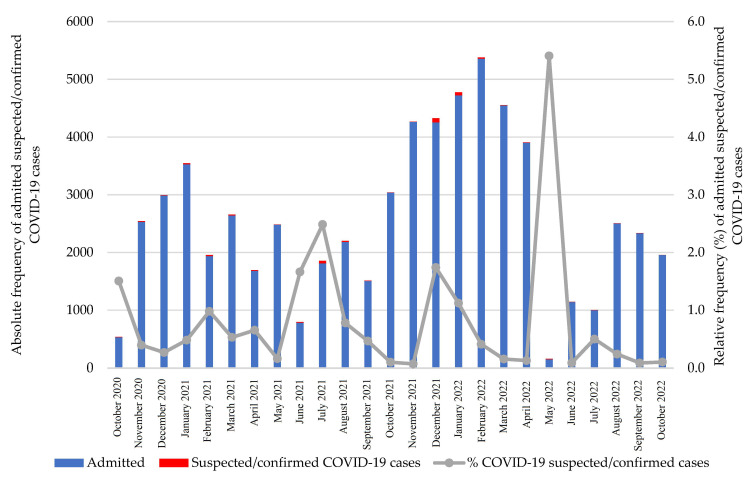
Monthly distribution of admitted suspected/confirmed COVID-19 cases by overall pediatric admission.

From October 2020 to October 2022, there were 63,753 admissions recorded at Hospital Central de Maputo pediatric ward. Among these, 364 (0.6%; 95% CI: 0.5–0.6) were COVID-19 suspected/confirmed cases. There were 6807 overall hospitalizations, of which 173 were COVID-19-positive cases (2.5%; 95% CI: 2.2–2.9). The monthly distribution of suspected/confirmed COVID-19 cases ranged from 0.1% to 5.4% (Figure 1).

**Figure 2:**
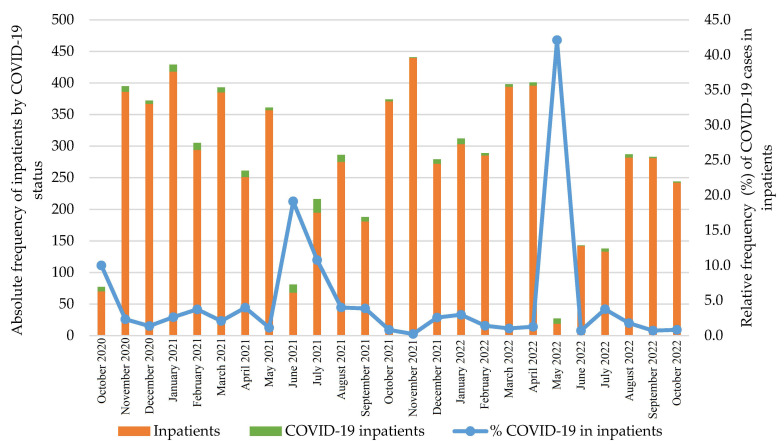
Monthly distribution of confirmed COVID-19 inpatients by overall pediatric inpatients.

The monthly distribution of hospitalized COVID-19-positive cases ranged from 0.2% to 42.1% (Figure 2). The hospitalization rate of the suspected/confirmed COVID-19 cases was 47.5% (95% CI: 42.4–52.7; 173/364).

### 3.2. Characteristics of Pediatric Children Hospitalized with COVID-19

Of the 173 children hospitalized with COVID-19, individual records were retrieved for 136 (78.6%). On admission, the most frequently reported clinical symptoms were difficulty in breathing (72.1%; 98/136), vomiting (51.5%; 70/136), and diarrhea (43.4%; 59/136; Table 1).

**Figure 3:**
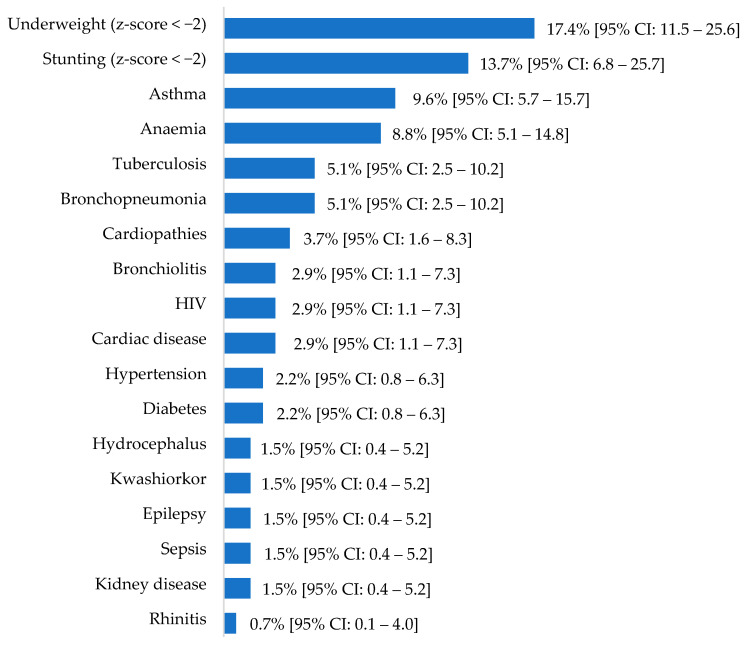
Relative morbidities with 95% CI in pediatric hospitalized cases of COVID-19 in the Hospital Central de Maputo.

In children hospitalized with COVID-19, being underweight was the most common comorbidity (17.4%; 19/109), followed by stunting (13.7%; 7/51; Figure 3). Additional conditions such as trauma, digestive hemorrhage, candidiasis, malaria, hematic syndrome, marasmus, meningocephaly, rhetynoblasma, Karel syndrome, cerebral affection, and dermatitis were observed with a frequency of 0.7% (95% CI: 0.1–4.0; 1/136).

Of the children hospitalized with COVID-19, 30.0% (95% CI: 22.2–39.1; 33/110) died in the Hospital Central de Maputo. Factors associated with mortality were difficulty in breathing, diarrhea, vomiting, convulsions, being underweight, and having received artificial support for breathing (*p*-value < 0.05; Table 2).

## 4. Discussion

Between October 2020 and October 2022, the COVID-19 burden in Mozambican children aged 0–168 months at the Hospital Central de Maputo was below 3%. In contrast to our findings, a previous hospital-based study in the same setting observed a burden of 37.3% [12], possibly because the previous study was implemented during the COVID-19 second wave, a period with an increased number of cases. Furthermore, the analysis was restricted to infants (1–12 months of age) in the breastfeeding ward and reported data from January and February 2021, while our data accounted for two consecutive years and included children from 0 to 168 months.

Peaks in admission converge with the increased number of cases during the waves. The highest number of admissions occurred during the wave in which the circulation of the Delta variant was reported in Mozambique [15], suggesting that morbidity depends on variant. In Pakistan, the Delta variant was associated with increased death rates in children aged up to 18 years [9].

School closure was implemented as a non-pharmacological measure to mitigate the spread of the virus and potentially reduce the number of COVID-19 cases requiring hospitalization in Mozambique. Our findings, however, suggest that morbidity was predominantly observed in non-school-aged children (less then five years old), indicating that school closures may have had a limited impact on reducing morbidity in school-aged children. It is important to note that regional differences may exist within Africa regarding the distribution of COVID-19 cases among different age groups. For instance, the overall median age in our study population was 5.9 years. However, in specific regions such as Eastern Africa (9.0 years), Western Africa (6.0 years), Central Africa (14 years), and Southern Africa (2.7 years), median ages varied significantly [10].

Our observed death rate (30%) among COVID-19 cases was notably higher than that in a previous study conducted in the same setting in infants (5.4%) [12]. Even when compared to other African countries, such as the Democratic Republic of Congo, Ghana, Kenya, South Africa, and Uganda, our mortality rate remained considerably higher, with our study reporting a mortality rate of 30.0% compared to an average of 8.3% (ranging from 5.3% to 14.0% between countries) [10]. Additionally, when compared to non-African countries, our mortality rate was higher than those reported in Pakistan (18.3%) [9], Brazil (8.7%) [16], the United Arab Emirates (0.0%) [17], and the United States of America (0.0–2.0%) [18,19,20,21].

In our sample, we observed that underweight was the most common comorbidity in hospitalized children with COVID-19 and was also associated with death. In contrast to high-income countries, overweight was more commonly associated with severe COVID-19 outcomes [11,18]. Being overweight was justified by enclosure measurements [22], which reduced physical activity and increased processed food consumption [23].

We observed a 17.4% proportion of underweight in hospitalized children with COVID-19. A representative national survey in the community in 2022-2023 identified a prevalence of 15.4% for underweight among healthy Mozambican children under five years old. In the same survey, in Maputo City, where we conducted our study, the prevalence of underweight was 4.4% [24]. Potential reasons for the observed differences may be related to the age range used in the survey and our study; moreover, our study was implemented in a public quaternary hospital which receives patients from all over the country. However, we did not record participants’ provenance.

Specific pre-existing comorbidities have been consistently associated with a severe clinical profile and death in children with COVID-19, as documented in previous studies [4,6,9,10,12]. These comorbidities may compromise the immune defense system, reducing resistance to viral infection, and previous organ damage can further exacerbate the severity of the disease’s manifestations [7].

During the COVID-19 pandemic, reports on food insecurity and increases in food prices coupled with reduced household income were identified as predictors of increased cases of undernutrition [25]. These factors highlight the heightened vulnerability of children to undernutrition during times of crisis such as a pandemic. Furthermore, considering that undernourished children may face an elevated risk of mortality if they contract COVID-19, implementing preventive measures such as prioritizing COVID-19 immunization in undernourished children could potentially reduce hospital mortality rates.

Difficulty breathing, diarrhea, vomiting, and convulsions were symptoms presented at admission that were also related to death and were reported in other locations [19,20,21,26]. A low rate of artificial support for breathing was observed compared with other settings [19,21]. This disparity could be attributed to factors such as the availability of oxygen therapy equipment in the health facility [27] or patients not meeting the requirements for artificial support for breathing, such as a peripheral oxygenation saturation (SpO_2_) level lower than 94%.

Our study design was reliant on the availability of paper-based records. However, we encountered a significant limitation due to a seven-month gap in data availability, spanning from the first reported COVID-19-positive case in March 2020 to September 2020. This gap resulted from a fire incident in the archive department, which may have led to an underestimation or overestimation of the burden of COVID-19 in our study. In the present study, only one health facility was included, which is less than one percent of the country’s health facilities [27].

In this setting, COVID-19 cases were mostly observed in underweight children, and underweight was also associated with death. Underweight has an heterogenous distribution within Mozambique’s provinces [24], suggesting that higher mortality rates in children with underweight and COVID-19 can be expected in provinces that share the highest burden of underweight, such as Cabo Delgado and Zambézia.

## 5. Conclusions

Overall, pediatric suspected/confirmed COVID-19 cases accounted for less than three percent of both outpatients and inpatients, although there was monthly variability. More than half of the hospitalized pediatric COVID-19 cases involved children under five years old. Underweight emerged as the most prevalent comorbidity among children hospitalized with COVID-19, and the percentage of underweight children who died was significantly higher. Given the heightened mortality among undernourished children compared to non-undernourished children, pharmacological interventions such as COVID-19 vaccination, should be prioritized for this vulnerable population.

## Figures and Tables

**Table 1:** Demographic characteristics and symptoms on admission of hospitalized pediatric cases of COVID-19 at Hospital Central de Maputo, Mozambique.

Characteristics	n/N	% (95% CI)
Sex		
Male	74/135	54.8 (46.4–63.0)
Female	61/135	45.2 (37.0–53.6)
Age group		
<5 years	97/129	75.2 (67.1–81.8)
≥5 years	32/129	24.8 (18.2–32.9)
Age in months, median (Q1–Q3), min–max	24 (8–48) 1–168	
Fever (body temperature ≥ 38 °C)	25/130	19.2 (13.4–26.8)
Nasal congestion	38/133	28.6 (21.6–36.8)
Difficulty breathing	98/136	72.1 (64.0–78.9)
Diarrhea	59/136	43.4 (35.3–51.8)
Vomit	70/136	51.5 (43.1–59.7)
Convulsions/neurological disorder	37/136	27.2 (20.4–35.2)
Received artificial support for breathing	9/136	6.6 (3.5–12.1)

**Table 2:** Comparison of in-hospital mortality of children hospitalized with COVID-19 at Hospital Central de Maputo.

Characteristics		% (n/N)	*p*-Value
Sex	Male	30.0% (18/60)	0.945 ^a^
	Female	30.6% (15/49)	
Age in months, median (Q1–Q3) min–max	Died	26 (12.5–72) 1–168	0.765 ^b^
	Survived	24 (10–60) 1–168	
Fever (body temperature ≥ 38 °C)	Yes	46.7% (7/15)	0.115 ^c^
	No	24.4% (22/90)	
Nasal congestion	Yes	28.6% (8/28)	0.791 ^a^
	No	31.2% (25/80)	
Difficulty breathing	Yes	38.2% (29/76)	0.005 ^c^
	No	11.8% (4/34)	
Diarrhea	Yes	53.3% (24/45)	<0.001 ^a^
	No	13.8% (9/65)	
Vomit	Yes	52.8% (28/53)	<0.001 ^a^
	No	8.8% (5/57)	
Convulsions/neurological disorder	Yes	65.5% (19/29)	<0.001 ^a^
	No	17.3% (14/81)	
Received artificial support for breathing	Yes	88.9% (8/9)	<0.001 ^c^
	No	24.8% (25/101)	
Underweight (z-score < −2)	Yes	61.5% (8/13)	0.005 ^c^
	No	21.3% (16/75)	
Stunting (z-score < −2)	Yes	0.0% (0/6)	1.000 ^c^
	No	2.4% (1/42)	
Asthma	Yes	15.4% (2/13)	0.337 ^c^
	No	32.0% (31/97)	
Anemia	Yes	44.4% (4/9)	0.448 ^c^
	No	28.7% (29/101)	
Tuberculosis	Yes	33.3% (2/6)	1.000 ^c^
	No	29.8% (31/104)	
Broncho-pneumonia	Yes	0.0% (0/7)	0.100 ^c^
	No	32.0% (33/103)	

^a^ Pearson’s chi-square test, ^b^ Mann–Whitney U test; ^c^ Fisher’s exact test.

## Data Availability

Data used for this analysis can be requested from the Instituto Nacional de Saúde, Mozambique, through the corresponding author. Researchers interested in secondary analysis must submit a research proposal for consideration by the study investigators as well as by the Directorate for Research in Health and Well-Being. Upon approval, the requestor must sign a data use agreement.

## References

[1] WHO Coronavirus Disease (COVID-19) Pandemic. https://www.who.int/europe/emergencies/situations/covid-19.

[2] WHO Coronavirus Disease (COVID-19). https://www.who.int/health-topics/coronavirus.

[3] Viner R.M., Mytton O.T., Bonell C., Melendez-Torres G.J., Ward J., Hudson L., Waddington C., Thomas J., Russell S., van der Klis F. (2021). Susceptibility to SARS-CoV-2 Infection Among Children and Adolescents Compared with Adults: A Systematic Review and Meta-Analysis. JAMA Pediatr..

[4] Solanki G., Wilkinson T., Bansal S., Shiba J., Manda S., Doherty T. (2022). COVID-19 Hospitalization and Mortality and Hospitalization-Related Utilization and Expenditure: Analysis of a South African Private Health Insured Population. PLoS ONE.

[5] Ho M., Most Z.M., Perl T.M., Diaz M.I., Casazza J.A., Saleh S., Pickering M., Radunsky A.P., Hanna J.J., Thakur B. (2023). Incidence and Risk Factors for Severe Outcomes in Pediatric Patients With COVID-19. Hosp. Pediatr..

[6] Tsankov B.K., Allaire J.M., Irvine M.A., Lopez A.A., Sauvé L.J., Vallance B.A., Jacobson K. (2021). Severe COVID-19 Infection and Pediatric Comorbidities: A Systematic Review and Meta-Analysis. Int. J. Infect. Dis..

[7] Zhang J., Dong X., Liu G., Gao Y. (2022). Risk and Protective Factors for COVID-19 Morbidity, Severity, and Mortality. Clin. Rev. Allerg. Immunol..

[8] Clinical Characteristics and Health Outcomes for Children Hospitalized with COVID-19. https://www.who.int/publications-detail-redirect/9789240080119.

[9] Abbas Q., Khalid F., Shahbaz F.F., Khan J., Mohsin S., Gowa M.A., Shaikh A.S., Asghar R.M., Khalid J., Karim S. (2023). Clinical and Epidemiological Features of Pediatric Population Hospitalized with COVID-19: A Multicenter Longitudinal Study (March 2020–December 2021) from Pakistan. Lancet Reg. Health Southeast Asia.

[10] Nachega J.B., Sam-Agudu N.A., Machekano R.N., Rabie H., Van Der Zalm M.M., Redfern A., Dramowski A., O’Connell N., Pipo M.T., Tshilanda M.B. (2022). Assessment of Clinical Outcomes Among Children and Adolescents Hospitalized With COVID-19 in 6 Sub-Saharan African Countries. JAMA Pediatr..

[11] Antoon J.W., Grijalva C.G., Thurm C., Richardson T., Spaulding A.B., Ii R.J.T., Reyes M.A., Shah S.S., Burns J.E., Kenyon C.C. (2021). Factors Associated With COVID-19 Disease Severity in US Children and Adolescents. J. Hosp. Med..

[12] Osório D., Liasse S.T., Cassia U., Sidat M., Taunde S., Mate B., Pambo E., Mazivila O., Elias B., Lorenzoni C. (2023). Test Positivity and Clinical Presentation of COVID-19 in Mozambican Infants Hospitalized during the Second Wave of the Pandemic in 2021. Pan Afr. Med. J..

[13] Braga J.M., Banze A.R., Dengo-Baloi L., Evaristo V.L., Rossetto E.V., Baltazar C.S. (2022). Investigation and Contact Tracing of the First Cases of COVID-19 in Mozambique, 2020. Pan Afr. Med. J..

[14] Brown L.D., Cai T.T., DasGupta A. (2001). Interval Estimation for a Binomial Proportion. Stat. Sci..

[15] Ismael N., van Wyk S., Tegally H., Giandhari J., San J.E., Moir M., Pillay S., Utpatel C., Singh L., Naidoo Y. (2023). Genomic Epidemiology of SARS-CoV-2 during the First Four Waves in Mozambique. PLoS Global Public Health.

[16] de Mello L.B., da Silva J.A., Clemente H.A., Neto J.A.B., Mello C.S. (2023). Nutritional Risk and Clinical Outcomes of COVID-19 in Hospitalized Children and Adolescents: A Multicenter Cohort. J. Pediatr..

[17] Al Dhaheri F.A., El Dannan H., Hashim M.J., Alshehi S., Al-Jburi F., Antali A., Al Jasmi N., Al Khouri S., Al Hajjar M., Abbas T. (2023). Clinical Outcomes of COVID-19 in Newborns and Infants: A Multicenter Experience of 576 Cases. Pediatr. Infect. Dis. J..

[18] Woodruff R.C., Campbell A.P., Taylor C.A., Chai S.J., Kawasaki B., Meek J., Anderson E.J., Weigel A., Monroe M.L., Reeg L. (2022). Risk Factors for Severe COVID-19 in Children. Pediatrics.

[19] Verma S., Lumba R., Dapul H.M., Gold-von Simson G., Phoon C.K., Lighter J.L., Farkas J.S., Vinci A., Noor A., Raabe V.N. (2021). Characteristics of Hospitalized Children with SARS-CoV-2 in the New York City Metropolitan Area. Hosp. Pediatr..

[20] Kim L., Whitaker M., O’Halloran A., Kambhampati A., Chai S.J., Reingold A., Armistead I., Kawasaki B., Meek J., Yousey-Hindes K. (2020). Hospitalization Rates and Characteristics of Children Aged <18 Years Hospitalized with Laboratory-Confirmed COVID-19—COVID-NET, 14 States, March 1–July 25, 2020. MMWR Morb. Mortal. Wkly. Rep..

[21] Kainth M.K., Goenka P.K., Williamson K.A., Fishbein J.S., Subramony A., Barone S., Belfer J.A., Feld L.M., Krief W.I., Palumbo N. (2020). Early Experience of COVID-19 in a US Children’s Hospital. Pediatrics.

[22] Chang T.-H., Chen Y.-C., Chen W.-Y., Chen C.-Y., Hsu W.-Y., Chou Y., Chang Y.-H. (2021). Weight Gain Associated with COVID-19 Lockdown in Children and Adolescents: A Systematic Review and Meta-Analysis. Nutrients.

[23] Woo S., Yang H., Kim Y., Lim H., Song H.J., Park K.H. (2022). Sedentary Time and Fast-Food Consumption Associated With Weight Gain During COVID-19 Lockdown in Children and Adolescents With Overweight or Obesity. J. Korean Med. Sci..

[24] Instituto Nacional de Estatística (INE) e ICF (2023). Inquérito Demográfico e de Saúde Em Moçambique 2022–2023.

[25] Bliznashka L., Ahun M.N., Velthausz D., Donco R., Karuskina-Drivdale S., Pinto J., Yousafzai A.K., Jeong J. (2022). Effects of COVID-19 on Child Health Services Utilization and Delivery in Rural Mozambique: A Qualitative Study. Health Policy Plan..

[26] van der Zalm M.M., Lishman J., Verhagen L.M., Redfern A., Smit L., Barday M., Ruttens D., da Costa A., van Jaarsveld S., Itana J. (2020). Clinical Experience With Severe Acute Respiratory Syndrome Coronavirus 2–Related Illness in Children: Hospital Experience in Cape Town, South Africa. Clin. Infect. Dis..

[27] Denhard L., Kaviany P., Chicumbe S., Muianga C., Laisse G., Aune K., Sheffel A. (2021). How Prepared Is Mozambique to Treat COVID-19 Patients? A New Approach for Estimating Oxygen Service Availability, Oxygen Treatment Capacity, and Population Access to Oxygen-Ready Treatment Facilities. Int. J. Equity Health.

